# Increased Biological Effective Dose of Radiation Correlates with Prolonged Survival of Patients with Limited-Stage Small Cell Lung Cancer: A Systematic Review

**DOI:** 10.1371/journal.pone.0156494

**Published:** 2016-05-26

**Authors:** Lucheng Zhu, Shirong Zhang, Xiao Xu, Bing Wang, Kan Wu, Qinghua Deng, Bing Xia, Shenglin Ma

**Affiliations:** 1 Affiliated Hangzhou Hospital of Nanjing Medical University, Hangzhou 310006, PR China; 2 Hangzhou First People’s Hospital, Hangzhou 310006, PR China; 3 HangZhou Cancer Hospital, Hangzhou 310006, PR China; Peking University People Hospital, CHINA

## Abstract

**Objective:**

Thoracic radiotherapy (TRT) is a critical component of the treatment of limited-stage small cell lung cancer (LS-SCLC). However, the optimal radiation dose/fractionation remains elusive. This study reviewed current evidence and explored the dose-response relationship in patients with LS-SCLC who were treated with radiochemotherapy.

**Materials and Methods:**

A quantitative analysis was performed through a systematic search of PubMed, Web of Science, and the Cochrane Library. The correlations between the biological effective dose (BED) and median overall survival (mOS), median progression-free survival (mPFS), 1-, 3-, and 5-year overall survival (OS) as well as local relapse (LR) were evaluated.

**Results:**

In all, 2389 patients in 19 trials were included in this study. Among these 19 trials, seven were conducted in Europe, eight were conducted in Asia and four were conducted in the United States. The 19 trials that were included consisted of 29 arms with 24 concurrent and 5 sequential TRT arms. For all included studies, the results showed that a higher BED prolonged the mOS (R^2^ = 0.198, p<0.001) and the mPFS (R^2^ = 0.045, p<0.001). The results also showed that increased BED improved the 1-, 3-, and 5-year OS. A 10-Gy increment added a 6.3%, a 5.1% and a 3.7% benefit for the 1-, 3-, and 5-year OS, respectively. Additionally, BED was negatively correlated with LR (R^2^ = 0.09, p<0.001). A subgroup analysis of concurrent TRT showed that a high BED prolonged the mOS (p<0.001) and the mPFS (p<0.001), improved the 1-, 3-, and 5-year OS (p<0.001) and decreased the rate of LR (p<0.001).

**Conclusion:**

This study showed that an increased BED was associated with improved OS, PFS and decreased LR in patients with LS-SCLC who were treated with combined chemoradiotherapy, which indicates that the strategy of radiation dose escalation over a limited time frame is worth exploring in a prospective clinical trial.

## Introduction

Small cell lung cancer (SCLC) accounts for 15% of all lung cancers. The two-drug combination of etoposide and cisplatin remains the standard of care for SCLC [1]. Limited-stage small cell lung cancer (LS-SCLC), which is defined as disease that can be encompassed within reasonable radiotherapy fields, accounts for one-third of all cases of SCLC at diagnosis [2]. Concurrent chemo-radiotherapy (CRT) is the standard treatment for LS-SCLC and is widely used in clinical practice [3,4]. CRT, along with prophylactic cranial irradiation (PCI), has yielded a wide-ranging median overall survival (OS) of 15.5 to 28.5 months [5,6].

Thoracic radiotherapy (TRT) is a critical component of the treatment of LS-SCLC. The efficacy of TRT is closely related to the radiation dose, but this treatment strategy is more complicated in clinical practice. Currently, total doses that range from 45–50 Gy have been used in order to limit toxicity but maintain the efficacy [7]. Previous data have indicated that doses of 45–50 Gy are associated with a high rate of locoregional failure, and a dose-response is still seen at doses ≥50 Gy, which suggests the feasibility of high-dose radiation [8,9]. Attempts have been made to administer high-dose radiation, but unfortunately, CALGB 39808, 30002 and 30202 failed to demonstrate the superiority of high-dose (70 Gy) radiation [10]. Some experts ascribed this to the prolonged overall radiation time (ORT) of the 70 Gy dose. In fact, several factors including single dose, fraction scheme, as well as the ORT may have an impact on the outcome.

The biological effective dose (BED) is based on tissue/tumor radiosensitivity, dose per fraction, total number of doses and the ORT [11–13]. Because it is a combination of both physical and radiobiologic factors, the BED could reflect the amount of lethal damage to a given tissue at an established dose over time and its biological response [14]. Because the transformation to the BED allows for the comparison of different radiation schedules, the BED is now widely used in the assessment of radiotherapy [15]. TRT plays an important role in the treatment of LS-SCLC, but the optimal radiation dose/fraction remains elusive. This study reviewed current evidence and explored the dose-response relationship within the perspective of BED.

## Materials and Methods

### Publication Search

We searched PubMed, Web of Science, and the Cochrane Library for trials that were published from 1966 to Aug 25, 2015. The search strategy used both MeSH terms and free-text words to increase sensitivity. The following search terms were used: “limited stage/limited disease”, “small cell lung cancer/carcinoma”, “LS-SCLC/LD-SCLC”, and “radiotherapy/radiation”. This study was approved by the Ethics Committee of Hangzhou First People’s Hospital.

### Inclusion and Exclusion criteria

The inclusion criteria were as follows: (1) Prospective studies ≥1 group containing TRT for LS-SCLC; (2) To minimize heterogeneity, we only included studies in which the chemotherapy regimen comprised etoposide and cisplatin (EP) or etoposide and carboplatin (EC); (3) Studies that reported the local relapse (LR), overall survival (OS), and progression-free survival (PFS). The exclusion criteria were as follows: (1) Patients who responded only to chemotherapy but who also received TRT; (3) Letters, editorials, expert opinions, case reports and reviews; (4) Studies without usable data; (5) Duplicate publications.

### Data extraction

Two investigators independently extracted the data from the eligible studies, and disagreements were resolved by discussion with a third investigator. For each study, the following information was recorded: the first author, year of publication, number of patients, dose per fraction, total dose, ORT, LR, PFS, and OS.

### Statistical analysis

To examine the dose-response relationship between the BED and LR, OS, as well as PFS, linear regression analyses weighted by sample size were used. For the 1- to 5-year OS rate, the following methods were used to extract the data: (1) obtained directly from the publication; (2) calculated and retrieved from Kaplan-Meier Curves. For the median OS and median PFS (mOS and mPFS), the above-mentioned methods were also used. Statistical analysis was performed with the IBM SPSS version 22.0 software. *P* values less than 0.05 were considered statistically significant. For the calculation of the BED, the following formulas were used [16]:

$$BED\;=\;nd\;*\;(1+\frac{d}{\alpha /\beta })-0.693\;*\frac{t}{\alpha T_{pot}}$$

Where ***α/β*** = 10, ***α*** = 0.3, ***T***_***pot***_ = potential doubling time (5.6 days), ***n*** represents the total number of fractions, ***d*** represents the dose per fraction, ***t*** represents the total radiation time (days).

## Results

### Characteristics of included studies

**Fig 1:**
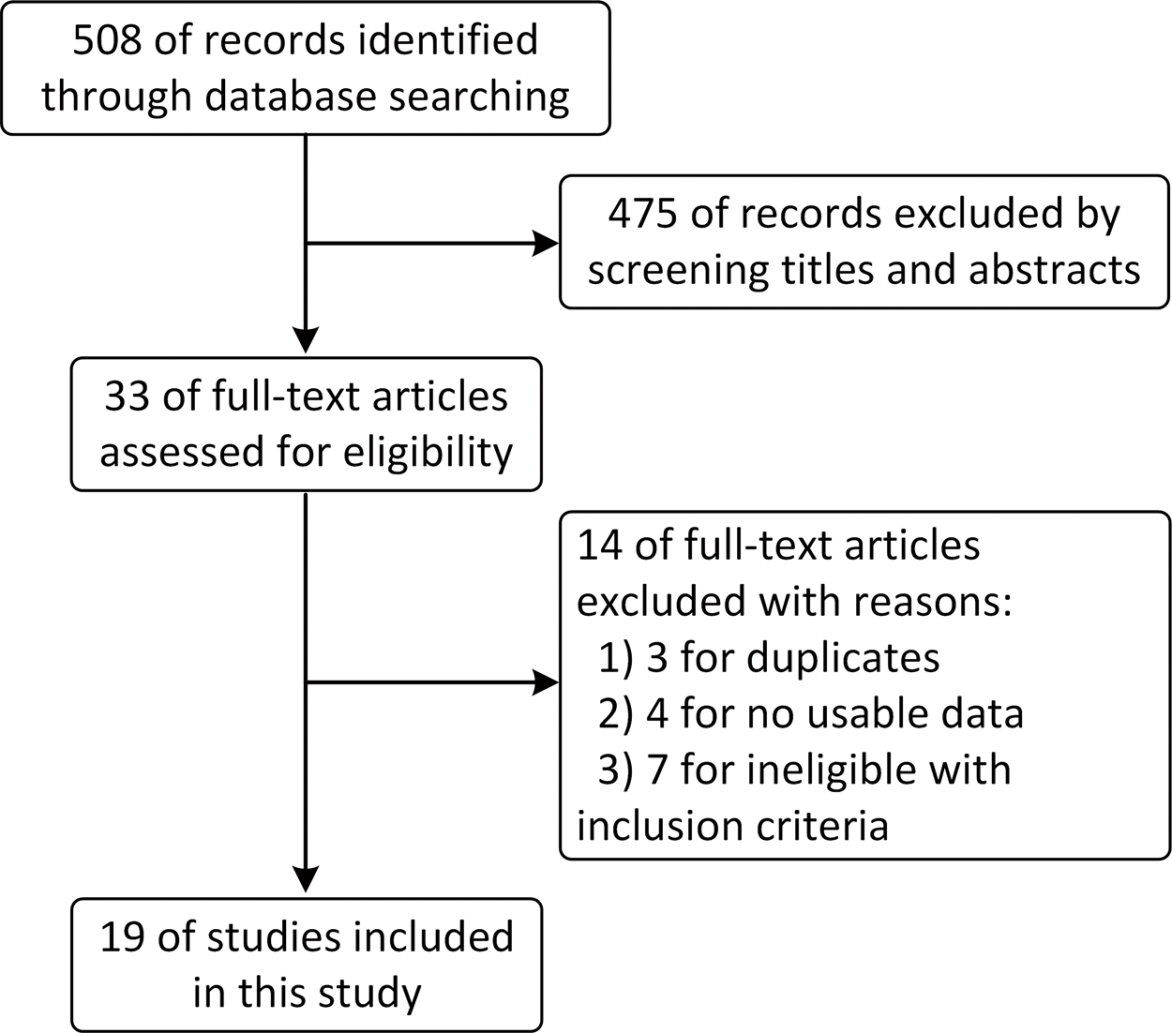
Flowchart of this systematic review.

As shown in Fig 1, the electronic search yielded 508 records. After the titles and abstracts were screened, 33 full-text articles were assessed for eligibility. Finally, 19 articles [5,6,17–33] were included in this systematic review. Among these 19 trials, seven were conducted in Europe, eight were conducted in Asia and four were conducted in the United States. These 19 trials consisted of 29 arms with 24 concurrent and 5 sequential radiochemotherapy arms. In regards to chemotherapy, 4 studies had 7 EC-based arms and 15 studies had 22 EP-based arms. These studies are summarized in detail in Table 1.

**Table 1:** Characteristics of 19 included studies.

Study	Year	Country	Phase	N	TRT	Manner	BED	Cycle Start RT	Chemotherapy	mOS (months)	mPFS (months)	LR (%)
Gronberg[17]	2015	Norway	II	84	42Gy/15F/15d	Concurrent	45.9	2	cisplatin 75 mg/m2 day1, etoposide 100 mg/m2 days1-3; 21-day cycle	18.8	10.2	34
				73	45Gy/30F/15d	Concurrent	43.9	2	cisplatin 75 mg/m2 day1, etoposide 100 mg/m2 days1-3; 21-day cycle	25.1	11.4	50
Xia[5]	2015	China	II	59	55Gy/22F/22d	Concurrent	56.4	2/3	cisplatin 25 mg/m2 days1-3, etoposide 70 mg/m2 days1-4; 21-day cycle	28.5	19	15
Sun[18]	2013	Korea	III	113	52.5Gy/25F/25d	Concurrent	49.9	1	cisplatin 70 mg/m2 day1, etoposide 100 mg/m2 days1-3; 21-day cycle	24.1	12.4	36.9
				109	52.5Gy/25F/25d	Concurrent	49.9	3	cisplatin 70 mg/m2 day1, etoposide 100 mg/m2 days1-3; 21-day cycle	26.8	11.2	48.1
Hu[19]	2012	China	II	43	45Gy/30F/15d	Concurrent	43.9	3	cisplatin 80 mg/m2 day1, etoposide 100 mg/m2 days1-3; 21-day cycle	25.4	19.4	28.6
Colaco[20]	2012	England	II	26	66Gy/33F/33d	Concurrent	60.6	2	cisplatin 60 mg/m2 day1, etoposide 120 mg/m2 days1-3; 21-day cycle	-	-	15.3
				12	45Gy/30F/15d	Concurrent	43.9	2	cisplatin 60 mg/m2 day1, etoposide 120 mg/m2 days1-3; 21-day cycle	-	-	40
Komaki[32]	2012	USA	II	71	61.2Gy/34F/34d	Concurrent	58.6	2	cisplatin 60 mg/m2 day1, etoposide 120 mg/m2 days1-3; 21-day cycle	19	9.9	27
Sculier[6]	2008	France	III	104	40Gy/15F/15d	Concurrent	42.7	1	cisplatin 90 mg/m2 day1, etoposide 100 mg/m2 days1-3; 21-day cycle	15.5	10.3	16
				100	40Gy/15F/15d	Concurrent	42.7	1	cisplatin 6 mg/m2 days1-5, 8–12, 15–19, etoposide 100 mg/m2 days1-3; 21-day cycle	17	10.1	19
De Ruysscher[21]	2006	Netherlands	II	27	45Gy/30F/15d	Concurrent	43.9	1	carboplatin AUC5 day1, etoposide 120 mg/m2 days1-3; 21-day cycle	21	16	26
McClay[22]	2005	USA	III	154	50Gy/25F/25d	Concurrent	46.4	4	cisplatin 80 mg/m2 day1, etoposide 80 mg/m2 days1-3; 21-day cycle	20.6	12.3	-
Chen[23]	2005	China	II	57	56Gy/40F/20d	Sequential	53.1	2 to 4	cisplatin 25–30 mg/m2 days1-3, etoposide 50–70 mg/m2 days1-3; 21-day cycle	24	-	27
Qiao[24]	2004	China	NR	45	60Gy/30F/30d	Concurrent	55.5	1	carboplatin 100 mg days1-5, etoposide 100 mg days1-5; 21-day cycle	26	-	24.2
				45	60Gy/30F/30d	Sequential	55.5	5	carboplatin 100 mg days1-5, etoposide 100 mg days1-5; 21-day cycle	19	-	39.5
Schild[33]	2004	USA	III	131	50.4Gy/28F/28d	Concurrent	43.0	4	cisplatin 30 mg/m2 days1-3, etoposide 130 mg/m2 days1-3; 28-day cycle	20.6	-	34
Takada[25]	2002	Japan	III	114	45Gy/30F/15d	Concurrent	43.9	1	cisplatin 80 mg/m2 day1, etoposide 100 mg/m2 days1-3; 28-day cycle	27.2	11.9	18
				114	45Gy/30F/15d	Sequential	43.9	4	cisplatin 80 mg/m2 day1, etoposide 100 mg/m2 days1-3; 28-day cycle	19.7	10.2	18
Sundstrom[26]	2002	Norway	III	105	42Gy/15F/15d	Concurrent	45.9	4	cisplatin 75 mg/m2 day1, etoposide 100 mg/m2 day1,oral etoposide 200 mg/m2 days2-4; 21-day cycle	14.5	-	-
Skarlos[27]	2001	Greece	II	42	45Gy/30F/15d	Concurrent	43.9	1	carboplatin AUC6 days1-3, etoposide 100 mg days1-3; 21-day cycle	17.5	9.5	-
				39	45Gy/30F/15d	Concurrent	43.9	4	carboplatin AUC6 days1-3, etoposide 100 mg days1-3; 21-day cycle	17	10.5	-
Turrisi[28]	1999	England	III	206	45Gy/25F/25d	Concurrent	39.5	1	cisplatin 60 mg/m2 day1, etoposide 120 mg/m2 days1-3; 21-day cycle	19	-	52
				211	45Gy/30F/15d	Concurrent	43.9	1	cisplatin 60 mg/m2 day1, etoposide 120 mg/m2 days1-3; 21-day cycle	23	-	36
Luo[29]	1999	China	NR	48	50Gy/25F/25d	Sequential	46.4	1	cisplatin 20 mg/m2 days1-5, etoposide 100 mg/m2 days1-3; 21/28-day cycle	-	-	33.3
				46	50Gy/25F/25d	Sequential	46.4	6	cisplatin 20 mg/m2 days1-5, etoposide 100 mg/m2 days1-3; 21/28-day cycle	-	-	43.5
Jeremic[30]	1997	Japan	NR	52	54Gy/36F/18d	Concurrent	52.5	0	cisplatin 30 mg/m2 days1-3, etoposide 120 mg/m2 days1-3; 21-day cycle	34	-	6
				51	54Gy/36F/18d	Concurrent	52.5	3	cisplatin 30 mg/m2 days1-3, etoposide 120 mg/m2 days1-3; 21-day cycle	26	-	26
Bunn[31]	1995	USA	III	108	45Gy/25F/25d	Concurrent	39.5	1	cisplatin 25 mg/m2 days1-3, etoposide 60 mg/m2 days1-3; 21-day cycle	17	-	-

***BED*** biological effective dose, ***mOS*** median overall survival, ***mPFS*** median progression-free survival, ***LR*** local-relapse rate, ***F*** fraction, ***d*** day, ***AUC*** area under the curve, ***NR*** not reported

#### Linear regression analysis of all included studies

**Fig 2:**
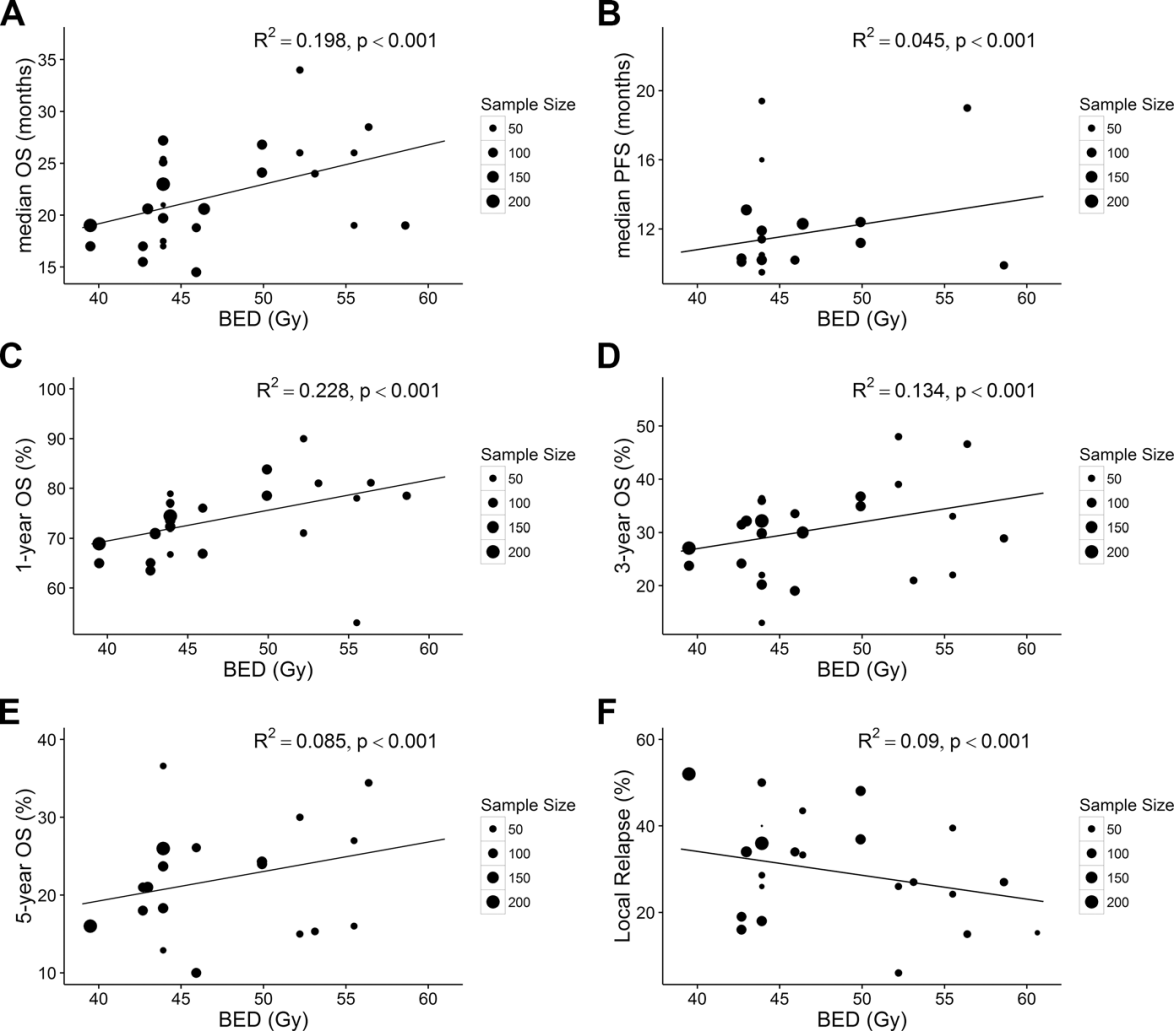
**Association between BED and mOS (A), mPFS (B), 1-year OS (C), 3-year OS (D), 5-year OS (E), and LR (F) for all included studies.** Each point in the plot represents a value of one arm. The point size represents the sample size. All analyses were conducted by linear regression methods weighted by sample size. ***BED***, biological effective dose; ***mOS***, median overall survival; **1-, 3-, 5-year *OS***, 1-, 3, 5-year overall survival; ***LR***, local relapse

In all, 2389 patients in 19 trials were included in this study. Twenty-five of 29 arms and 15 of 29 arms reported the mOS and mPFS, respectively. The mOS and mPFS ranged from 14.5 to 34 months and from 9.5 to 19.4 months, respectively. The results showed that a higher BED prolonged the mOS (R^2^ = 0.198, p<0.001) and the mPFS (R^2^ = 0.045, p<0.001) (Fig 2A and 2B). In order to explore the impact of the BED on long-term survival, we analyzed the correlation between the BED with the 1-, 3-, and 5-year OS. This analysis showed that an increased BED improved the 1-, 3-, and 5-year OS (R^2^ = 0.228, p<0.001; R^2^ = 0.134, p<0.001; R^2^ = 0.085, p<0.001, respectively) (Fig 2C–2E). A 10-Gy increment added a 6.3%, a 5.1% and a 3.7% benefit with respect to the 1-, 3-, and 5-year OS, respectively. Previous data indicated that a total dose of 45–50 Gy was still associated with a high rate of locoregional failure, but whether an increased BED could decrease the LR rate was unknown. Therefore, we further analyzed the relationship between the BED and LR. The results showed that the BED was correlated with the incidence of LR (R^2^ = 0.09, p<0.001)(Fig 2F). A 10-Gy increment in the BED could decrease the LR rate by 7.1%. As significant heterogeneity was present across the included studies (small R^2^ value), we next performed subgroup analyses.

### Analysis of concurrent chemo-radiotherapy

**Fig 3:**
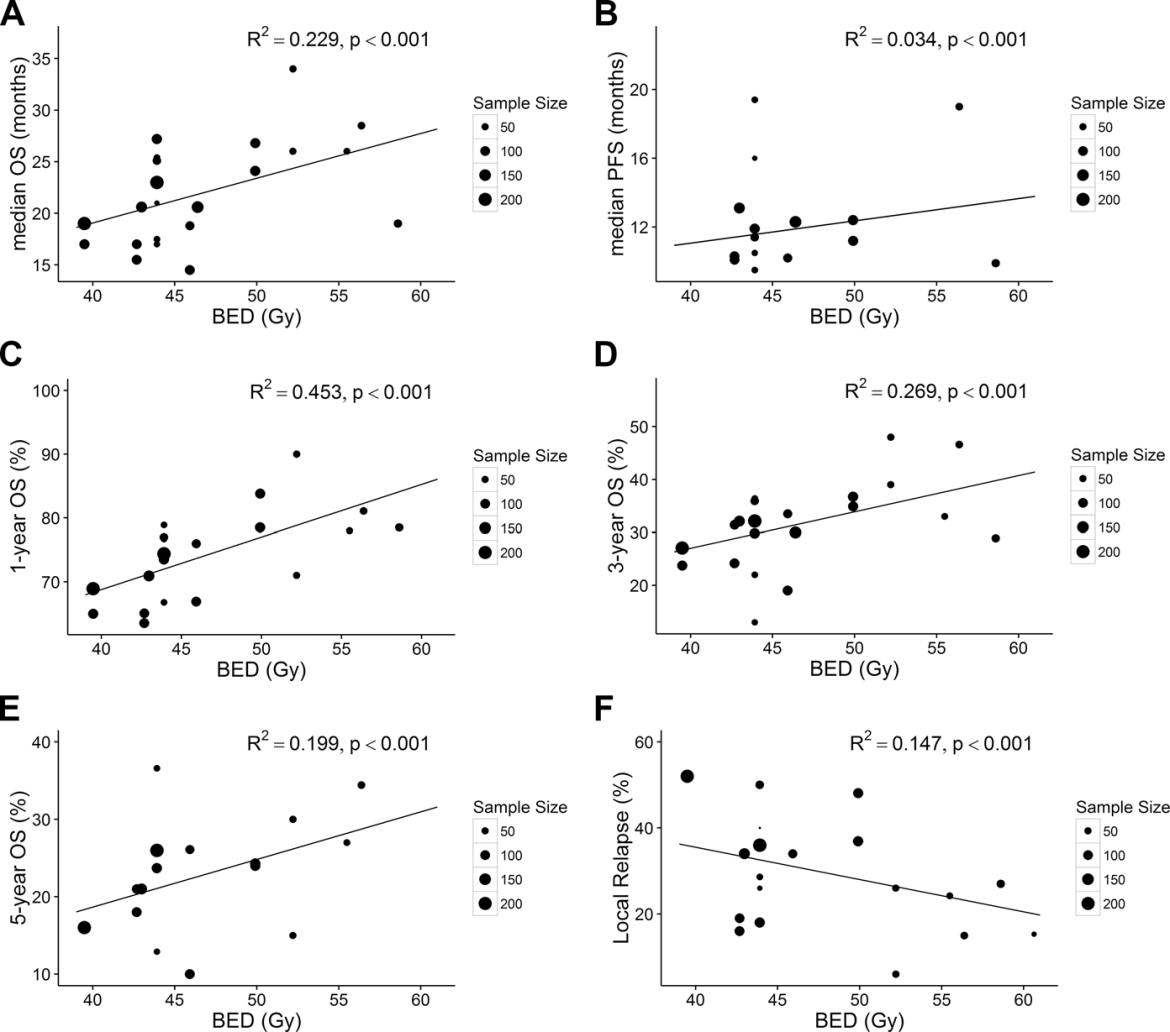
**Association between BED and mOS (A), mPFS (B), 1-year OS (C), 3-year OS (D), 5-year OS (E), and LR (F) in the setting of concurrent chemo-radiotherapy.** Each point in the plot represents a value of one arm. The point size represents the sample size. All analyses were conducted by linear regression methods weighted by sample size. ***BED***, biological effective dose; ***mOS***, median overall survival; **1-, 3-, 5-year *OS***, 1-, 3, 5-year overall survival; ***LR***, local relapse

Previous studies have indicated that concurrent chemo-radiotherapy is one of the optimal medical treatments for LS-SCLC. Therefore, we conducted this analysis to explore the dose-response relationship of the BED in the setting of concurrent chemo-radiotherapy. After the removal of 3 studies that reported only sequential chemo-radiotherapy [23,24,29], 2041 patients from 16 studies were finally included in subsequent subgroup analyses. The result revealed that a higher BED could prolong the mOS (R^2^ = 0.229, p<0.001) and the mPFS (R^2^ = 0.034, p<0.001) (Fig 3A and 3B). According to our calculated model, an increase in the BED from 45 Gy to 60 Gy added 6.5 months to the mOS. Moreover, our data showed that an increased BED improved the 1-, 3-, and 5-year OS (Fig 3C and 3E) and decreased the rate of LR (R^2^ = 0.147, p<0.001)(Fig 3F).

## Discussion

This study reviewed current evidence and explored the potential of high dose TRT for the treatment of LS-SCLC. Our results showed that an increased BED prolonged the OS and PFS. A 10-Gy increment also added a 6.3%, a 5.1% and a 3.7% benefit with respect to the 1-, 3-, and 5-year OS, respectively. A retrospective study enrolled 205 LS-SCLC patients and compared the outcomes of a high BED (>57Gy) and a low BED (≤57Gy). That study demonstrated that the high BED (>57 Gy) group had a significantly better OS and PFS.

Currently, the combination of platinum and etoposide is the standard chemotherapy for LS-SCLC. A meta-analysis of four eligible trials showed no differences in efficacy between cisplatin and carboplatin as a first-line treatment for SCLC [34]. In order to minimize chemotherapy-induced effects, we only included trials that adopted the cisplatin/carboplatin plus etoposide regimen. This study features seven EC-based arms and twenty-two EP-based arms.

In the late 1950s, the Veterans Administration Lung Study Group (VALSG) defined LS-SCLC as a disease that is confined to the ipsilateral hemithorax that could be treated in a single radiation field [35]. This definition indirectly illustrated the important role of RT in LS-SCLC. First-line chemotherapy alone could achieve a response rate of 60–70%, whereas the combination of chemotherapy and TRT further improved overall survival [3,36,37]. Efforts that explore the optimal TRT schedule are currently underway. In 2002, JCOG9104 evaluated the efficacy of concurrent and sequential TRT for LS-SCLC and demonstrated that concurrent TRT was more effective than sequential TRT [25]. In this study, 5 arms of 4 trials involved sequential TRT, while 24 arms of 17 trials involved concurrent TRT. We found that the BED was positively correlated with OS in the setting of concurrent TRT (p<0.001). This suggested that a dose-response relationship still existed even in the case of a high BED (more than 45 Gy/30 F/15 d). A high BED strategy might be considered as an option in future trials.

Local treatment failure occurs in approximately 30% of LS-SCLC patients who are treated with the current standard therapy. LR is a major risk factor of survival. Our results showed that the BED was negatively correlated with LR (p<0.001). A 10Gy increment in the BED could decrease the LR rate by 5.5%. This suggested that a decrease in local failure might eventually contribute to a benefit in OS in patients who are treated with a high BED. However, RTOG 0617 demonstrated patients with non-small cell lung cancer receiving a high dose of 74Gy had a worse survival than 60Gy in the era of PET-CT staging and modern radiotherapy [38]. Increased dose might offer better local control as well as potential survival benefit, while the corresponding toxicity could not be ingored. RTOG0617 focused on the prevention of radiation pneumonitis in the design, whereas it underestimated the importance of heart protection. Analysis showed elevated heart dose in high dose group might have increased mortality. QOL analysis of the trial indicated IMRT provided better normal tissue protection [39], suggesting increase dose to SCLC might employ advanced radiation technique and value the importance of normal tissue protection in the future trials.

SCLC is characterized by a high invasion rate and rapid proliferation. Total dose-based measurements underestimate the effect of accelerated proliferation during a course of radiation, especially 30–40 days after the initial dose is given. CALGB 39808, 30002 and 30202 compared the efficacy of 45 Gy and 70 Gy radiation in patients with LS-SCLC, but these trials failed to demonstrate the superiority of high-dose (70 Gy) radiation [10]. This failure might be ascribed to prolonged ORT in the setting of 70 Gy radiation. Previous studies reported the T_pot_ of SCLC ranged from 2.6 to 8.6 days [40–42]. The high proliferation rate makes repopulation extremely important during the late course of therapy. The ORT of the included trials ranged from 3 to 7 weeks. Therefore, it was necessary to take the ORT into account.

Many researchers proposed models to calculate the BED for SCLC. Among these models, we agreed with the model proposed by *Fowler JF*. [16] which took ORT and tumor doubling time into consideration simultaneously and was also used in the study of *schild* [43]. Our previous study also adopted this model to investigate the biological radiation dose-response for Chinese patients [44]. T_pot_ of SCLC ranged from 2.6 to 8.6 days reported by previous publication. In this study, T_pot_ was set to 5.6 days which was same with the study of *schild*. *Schild et al*. found the Pearson correlation coefficient of 0.81 between BED and 5-year survival indicating a strong positive correlation. Although it was well performed, the authors only incorporated 8 studies and newer studies were not included as limitation of publication time. In addition, the parameters of LR and PFS were not analyzed in that study. Thus, it is of importance to perform this study.

However, R^2^ values were small in both the mPFS and LR analyses. This may be explained by the following reasons: (1) to obtain survival data, researchers usually followed-up patients periodically, and therefore, it was relatively easy to record these data especially in prospective studies; (2) The evaluation of LR and mPFS depended on imaging examinations. Whereas different studies adopted different techniques including chest radiography, computed tomography or positron emission tomography, these techniques varied in their sensitivities. In addition, survival data could be obtained by telephone or other communication tools, whereas the evaluation of the PFS and LR was limited to the hospital. Patient compliance inevitably had an impact on the evaluation.

Nevertheless, our study has several limitations. The timing of radiotherapy (early vs. later), the dose schedule of chemotherapy, radiation schedule (once daily vs. twice daily), and the types of radiation (conventional, 3-dimensional, intensity-modulated radiation therapy) all yielded different outcomes. Compared to daily fractionation radiation, twice daily fractionation radiation showed better survival benefit [17,20,28]. Although twice daily fractionation radiation overcomes the problem of tumor repopulation and re-oxygenation, inconvenience of this schedule and compliance of patients limits its use. Most of the included articles were single-arm phase II studies with small sample sizes, and thus our results were prone to be influenced by chance errors even though we had performed these calculations after they were weighted by sample size. Patients from different regions who participated in studies that were conducted at different research centers increased the inherent heterogeneity and might have influenced the validity of the results. Furthermore, the time span of included publications was as long as 20 years. Advances in radiation techniques and radiographic diagnosis as well as new staging systems and supportive treatments have the potential to provide survival benefits. Nevertheless, this study still had certain implications that might contribute to the exploration of an optimal TRT scheme for LS-SCLC.

In **conclusion**, we thought it was necessary to conduct this systematic review. This study explored the correlation of BED with mOS, mPFS, 1-, 3-, and 5-year OS and LR. The results showed that an increased BED could prolong survival and decrease LR. Compared with the current accepted radiation scheme (45 Gy/30 F/15 d), a higher BED might be a feasible strategy.

## Supporting Information

S1 File PRISMA checklist. (DOC)

## References

[1] Jett JR, Schild SE, Kesler KA, Kalemkerian GP (2013) Treatment of small cell lung cancer: Diagnosis and management of lung cancer, 3rd ed: American College of Chest Physicians evidence-based clinical practice guidelines. Chest 143: e400S–419S. 10.1378/chest.12-2363 23649448

[2] Govindan R, Page N, Morgensztern D, Read W, Tierney R, Vlahiotis A, et al (2006) Changing epidemiology of small-cell lung cancer in the United States over the last 30 years: analysis of the surveillance, epidemiologic, and end results database. J Clin Oncol 24: 4539–4544. 17008692 10.1200/JCO.2005.04.4859

[3] Pignon JP, Arriagada R, Ihde DC, Johnson DH, Perry MC, Souhami RL, et al (1992) A meta-analysis of thoracic radiotherapy for small-cell lung cancer. N Engl J Med 327: 1618–1624. 1331787 10.1056/NEJM199212033272302

[4] Rudin CM, Ismaila N, Hann CL, Malhotra N, Movsas B, Norris K, et al (2015) Treatment of Small-Cell Lung Cancer: American Society of Clinical Oncology Endorsement of the American College of Chest Physicians Guideline. J Clin Oncol 33: 4106–4111. 10.1200/JCO.2015.63.7918 26351333

[5] Xia B, Hong LZ, Cai XW, Zhu ZF, Liu Q, Zhao KL, et al (2015) Phase 2 study of accelerated hypofractionated thoracic radiation therapy and concurrent chemotherapy in patients with limited-stage small-cell lung cancer. Int J Radiat Oncol Biol Phys 91: 517–523. 10.1016/j.ijrobp.2014.09.042 25481679

[6] Sculier JP, Lafitte JJ, Efremidis A, Florin MC, Lecomte J, Berchier MC, et al (2008) A phase III randomised study of concomitant induction radiochemotherapy testing two modalities of radiosensitisation by cisplatin (standard versus daily) for limited small-cell lung cancer. Ann Oncol 19: 1691–1697. 10.1093/annonc/mdn354 18504252

[7] Carney DN, Mitchell JB, Kinsella TJ (1983) In vitro radiation and chemotherapy sensitivity of established cell lines of human small cell lung cancer and its large cell morphological variants. Cancer Res 43: 2806–2811. 6303568

[8] Roof KS, Fidias P, Lynch TJ, Ancukiewicz M, Choi NC (2003) Radiation dose escalation in limited-stage small-cell lung cancer. Int J Radiat Oncol Biol Phys 57: 701–708. 14529774 10.1016/s0360-3016(03)00715-6

[9] Movsas B, Moughan J, Komaki R, Choy H, Byhardt R, Langer C, et al (2003) Radiotherapy patterns of care study in lung carcinoma. J Clin Oncol 21: 4553–4559. 14597743 10.1200/JCO.2003.04.018

[10] Salama JK, Hodgson L, Pang H, Urbanic JJ, Blackstock AW, Schild SE, et al (2013) A pooled analysis of limited-stage small-cell lung cancer patients treated with induction chemotherapy followed by concurrent platinum-based chemotherapy and 70 Gy daily radiotherapy: CALGB 30904. J Thorac Oncol 8: 1043–1049. 10.1097/JTO.0b013e318293d8a4 23715301 PMC3822578

[11] Williams MV, Denekamp J, Fowler JF (1985) A review of alpha/beta ratios for experimental tumors: implications for clinical studies of altered fractionation. Int J Radiat Oncol Biol Phys 11: 87–96. 3881377 10.1016/0360-3016(85)90366-9

[12] Barendsen GW (1982) Dose fractionation, dose rate and iso-effect relationships for normal tissue responses. Int J Radiat Oncol Biol Phys 8: 1981–1997. 6759484 10.1016/0360-3016(82)90459-x

[13] Fowler JF (1989) The linear-quadratic formula and progress in fractionated radiotherapy. Br J Radiol 62: 679–694. 2670032 10.1259/0007-1285-62-740-679

[14] Dale RG, Jones B (1998) The clinical radiobiology of brachytherapy. Br J Radiol 71: 465–483. 9691890 10.1259/bjr.71.845.9691890

[15] Jones B, Dale RG, Deehan C, Hopkins KI, Morgan DA (2001) The role of biologically effective dose (BED) in clinical oncology. Clin Oncol (R Coll Radiol) 13: 71–81. 11373882 10.1053/clon.2001.9221

[16] Fowler JF (2001) Biological factors influencing optimum fractionation in radiation therapy. Acta Oncol 40: 712–717. 11765065 10.1080/02841860152619124

[17] Gronberg BH, Halvorsen TO, Flotten O, Brustugun OT, Brunsvig PF, Aasebo U, et al (2015) Randomized phase II trial comparing twice daily hyperfractionated with once daily hypofractionated thoracic radiotherapy in limited disease small cell lung cancer. Acta Oncol 30: 1–7. 10.3109/0284186X.2015.1092584 26494411

[18] Sun JM, Ahn YC, Choi EK, Ahn MJ, Ahn JS, Lee SH, et al (2013) Phase III trial of concurrent thoracic radiotherapy with either first- or third-cycle chemotherapy for limited-disease small-cell lung cancer. Ann Oncol 24: 2088–2092. 10.1093/annonc/mdt140 23592701

[19] Hu X, Bao Y, Zhang L, Guo Y, Chen YY, Li KX, et al (2012) Omitting elective nodal irradiation and irradiating postinduction versus preinduction chemotherapy tumor extent for limited-stage small cell lung cancer: interim analysis of a prospective randomized noninferiority trial. Cancer 118: 278–287. 10.1002/cncr.26119 21598237

[20] Colaco R, Sheikh H, Lorigan P, Blackhall F, Hulse P, Califano R, et al (2012) Omitting elective nodal irradiation during thoracic irradiation in limited-stage small cell lung cancer—evidence from a phase II trial. Lung Cancer 76: 72–77. 10.1016/j.lungcan.2011.09.015 22014897

[21] De Ruysscher D, Bremer RH, Koppe F, Wanders S, van Haren E, Hochstenbag M, et al (2006) Omission of elective node irradiation on basis of CT-scans in patients with limited disease small cell lung cancer: a phase II trial. Radiother Oncol 80: 307–312. 16949169 10.1016/j.radonc.2006.07.029

[22] McClay EF, Bogart J, Herndon JE 2nd, Watson D, Evans L, Seagren SL, et al (2005) A phase III trial evaluating the combination of cisplatin, etoposide, and radiation therapy with or without tamoxifen in patients with limited-stage small cell lung cancer: Cancer and Leukemia Group B Study (9235). Am J Clin Oncol 28: 81–90. 15685040 10.1097/01.coc.0000139940.52625.d0

[23] Chen GY, Jiang GL, Wang LJ, Qian H, Fu XL, Yang H, et al (2005) Cisplatin/etoposide chemotherapy combined with twice daily thoracic radiotherapy for limited small-cell lung cancer: a clinical phase II trial. Int J Radiat Oncol Biol Phys 61: 70–75. 15629596 10.1016/j.ijrobp.2004.04.058

[24] Qiao TK, Zhou DA, Xin L, Shu L, Wu W (2004) [Concurrent radiotherapy combined with carboplatin and etoposide in limited stage small cell lung cancer]. Zhonghua Jie He He Hu Xi Za Zhi 27: 237–239. 15144613

[25] Takada M, Fukuoka M, Kawahara M, Sugiura T, Yokoyama A, Yokota S, et al (2002) Phase III study of concurrent versus sequential thoracic radiotherapy in combination with cisplatin and etoposide for limited-stage small-cell lung cancer: results of the Japan Clinical Oncology Group Study 9104. J Clin Oncol 20: 3054–3060. 12118018 10.1200/JCO.2002.12.071

[26] Sundstrom S, Bremnes RM, Kaasa S, Aasebo U, Hatlevoll R, Dahle R, et al (2002) Cisplatin and etoposide regimen is superior to cyclophosphamide, epirubicin, and vincristine regimen in small-cell lung cancer: results from a randomized phase III trial with 5 years' follow-up. J Clin Oncol 20: 4665–4672. 12488411 10.1200/JCO.2002.12.111

[27] Skarlos DV, Samantas E, Briassoulis E, Panoussaki E, Pavlidis N, Kalofonos HP, et al (2001) Randomized comparison of early versus late hyperfractionated thoracic irradiation concurrently with chemotherapy in limited disease small-cell lung cancer: a randomized phase II study of the Hellenic Cooperative Oncology Group (HeCOG). Ann Oncol 12: 1231–1238. 11697833 10.1023/a:1012295131640

[28] Turrisi AT 3rd, Kim K, Blum R, Sause WT, Livingston RB, Komaki R, et al (1999) Twice-daily compared with once-daily thoracic radiotherapy in limited small-cell lung cancer treated concurrently with cisplatin and etoposide. N Engl J Med 340: 265–271. 9920950 10.1056/NEJM199901283400403

[29] Luo MH, Liu HB, Cao XL, Shi XF (1999) [Time and sequence of chemotherapy and radiotherapy in the treatment of lung cancer observation of 94 limited small cell lung cancer]. Chinese Journal of Radiation Oncology. pp. 222–224.

[30] Jeremic B, Shibamoto Y, Acimovic L, Milisavljevic S (1997) Initial versus delayed accelerated hyperfractionated radiation therapy and concurrent chemotherapy in limited small-cell lung cancer: a randomized study. J Clin Oncol 15: 893–900. 9060525 10.1200/JCO.1997.15.3.893

[31] Bunn PA Jr, Crowley J, Kelly K, Hazuka MB, Beasley K, Upchurch C, et al (1995) Chemoradiotherapy with or without granulocyte-macrophage colony-stimulating factor in the treatment of limited-stage small-cell lung cancer: a prospective phase III randomized study of the Southwest Oncology Group. J Clin Oncol 13: 1632–1641. 7602352 10.1200/JCO.1995.13.7.1632

[32] Komaki R, Paulus R, Ettinger DS, Videtic GM, Bradley JD, Glisson BS, et al (2012) Phase II study of accelerated high-dose radiotherapy with concurrent chemotherapy for patients with limited small-cell lung cancer: Radiation Therapy Oncology Group protocol 0239. Int J Radiat Oncol Biol Phys 83: e531–536. 10.1016/j.ijrobp.2012.01.075 22560543 PMC3377848

[33] Schild SE, Bonner JA, Shanahan TG, Brooks BJ, Marks RS, Geyer SM, et al (2004) Long-term results of a phase III trial comparing once-daily radiotherapy with twice-daily radiotherapy in limited-stage small-cell lung cancer. Int J Radiat Oncol Biol Phys 59: 943–951. 15234027 10.1016/j.ijrobp.2004.01.055

[34] Rossi A, Di Maio M, Chiodini P, Rudd RM, Okamoto H, Skarlos DV, et al (2012) Carboplatin- or cisplatin-based chemotherapy in first-line treatment of small-cell lung cancer: the COCIS meta-analysis of individual patient data. J Clin Oncol 30: 1692–1698. 10.1200/JCO.2011.40.4905 22473169

[35] Argiris A, Murren JR (2001) Staging and clinical prognostic factors for small-cell lung cancer. Cancer J 7: 437–447. 11693903

[36] Simon M, Argiris A, Murren JR (2004) Progress in the therapy of small cell lung cancer. Crit Rev Oncol Hematol 49: 119–133. 15012973 10.1016/S1040-8428(03)00118-5

[37] Arriagada R, Pignon JP, Ihde DC, Johnson DH, Perry MC, Souhami RL, et al (1994) Effect of thoracic radiotherapy on mortality in limited small cell lung cancer. A meta-analysis of 13 randomized trials among 2,140 patients. Anticancer Res 14: 333–335. 8166478

[38] Bradley JD, Paulus R, Komaki R, Masters G, Blumenschein G, Schild S, et al (2015) Standard-dose versus high-dose conformal radiotherapy with concurrent and consolidation carboplatin plus paclitaxel with or without cetuximab for patients with stage IIIA or IIIB non-small-cell lung cancer (RTOG 0617): a randomised, two-by-two factorial phase 3 study. Lancet Oncol 16: 187–199. 10.1016/S1470-2045(14)71207-0 25601342 PMC4419359

[39] Movsas B, Hu C, Sloan J, Bradley J, Komaki R, Masters G, et al (2016) Quality of Life Analysis of a Radiation Dose-Escalation Study of Patients With Non-Small-Cell Lung Cancer: A Secondary Analysis of the Radiation Therapy Oncology Group 0617 Randomized Clinical Trial. JAMA Oncol 2: 359–367. 10.1001/jamaoncol.2015.3969 26606200 PMC4786463

[40] Tinnemans MM, Schutte B, Lenders MH, Ten Velde GP, Ramaekers FC, Blijham GH (1993) Cytokinetic analysis of lung cancer by in vivo bromodeoxyuridine labelling. Br J Cancer 67: 1217–1222. 8512806 10.1038/bjc.1993.228 PMC1968512

[41] Kerr KM, Lamb D (1984) Actual growth rate and tumour cell proliferation in human pulmonary neoplasms. Br J Cancer 50: 343–349. 6087867 10.1038/bjc.1984.181 PMC1976798

[42] Shibamoto Y, Ike O, Mizuno H, Fukuse T, Hitomi S, Takahashi M (1998) Proliferative activity and micronucleus frequency after radiation of lung cancer cells as assessed by the cytokinesis-block method and their relationship to clinical outcome. Clin Cancer Res 4: 677–682. 9533537

[43] Schild SE, Bonner JA, Hillman S, Kozelsky TF, Vigliotti AP, Marks RS, et al (2007) Results of a phase II study of high-dose thoracic radiation therapy with concurrent cisplatin and etoposide in limited-stage small-cell lung cancer (NCCTG 95-20-53). J Clin Oncol 25: 3124–3129. 17634491 10.1200/JCO.2006.09.9606

[44] Xia B, Chen GY, Cai XW, Zhao JD, Yang HJ, Fan M, et al (2011) The effect of bioequivalent radiation dose on survival of patients with limited-stage small-cell lung cancer. Radiat Oncol 6: 50 10.1186/1748-717X-6-50 21592406 PMC3117707

